# Sexual harassment at medical schools: barriers to reporting and strategies for improvement - a nationwide, cross-sectional, multi-method study from Germany

**DOI:** 10.1136/bmjopen-2025-114374

**Published:** 2026-08-21

**Authors:** Maximilian Vogt, Michelle Förstel, Sabine Drossard

**Affiliations:** 1 Department of Neurosurgery, University Hospital Greifswald, Greifswald, Germany; 2 Department of Gynecology and Obstetrics, University Hospital Heidelberg, Heidelberg, Germany; 3 German Medical Students’ Association (bvmd), Berlin, Germany; 4 Department of General, Visceral, Transplant, Vascular and Pediatric Surgery, University Hospital Würzburg, Würzburg, Germany

**Keywords:** MEDICAL EDUCATION & TRAINING, Health policy, Occupational Health Services, Schools, Medical, Capacity Building, Cross-Sectional Studies

## Abstract

**Abstract:**

**Objectives:**

To examine the association between institutional reporting and support structures and the reporting behaviour, perceived support and satisfaction with the handling of sexual harassment among medical students in Germany.

**Design:**

Cross-sectional multi-method study combining a nationwide student survey with a structured survey of medical school deaneries and a structured documentary analysis of publicly available institutional information.

**Setting:**

All medical faculties offering a full undergraduate medical curriculum in Germany.

**Participants:**

A total of 5681 medical students from 44 faculties participated in an anonymous online survey (77.8% women). Additionally, 34 of 42 medical faculties (81.0%) responded to a structured questionnaire regarding institutional support and reporting structures. Publicly accessible documents and faculty websites were analysed using a structured documentary analysis.

**Primary and secondary outcome measures:**

Primary outcomes included (1) availability and visibility of reporting structures, (2) reporting of sexual harassment and (3) satisfaction with the reporting process. Secondary outcomes included perceived accessibility of reporting pathways, awareness of support structures, psychosocial support and perceived institutional consequences. Associations between student-reported outcomes, deanery responses and institutional characteristics were examined using multiple logistic and linear regression analyses.

**Results:**

Most medical faculties (88.2%) indicated the presence of a dedicated advisory service for medical students. However, only a minority provided online reporting forms (29.4%) or chat-based options (8.8%). Institutional guidelines were available at almost all (97.6%) faculties. 44.2% of students reported having experienced sexual harassment during medical training, but only 12.7% of affected students reported an incident. Nearly half of the affected students (48.5%) indicated that they did not know where or how to report. Reporting behaviour was strongly associated with the perceived psychological burden (OR 3.48; 95% CI 1.67 to 7.27), and secondarily with prior non-medical vocational experience or later stage of study. Satisfaction with the reporting process and willingness to report again in the future were positively associated with perceived consequences (β=0.49; p<0.001) and psychosocial support (β=0.35; p<0.001). Awareness and perceived accessibility of structures were particularly low among those affected.

**Conclusions:**

Most medical faculties provide counselling and reporting services, yet students, particularly those affected, often do not perceive them as visible, accessible or effective. We propose four additional strategies for medical faculties: (1) Awareness and Competence-Building, (2) Visibility and Active Communication, (3) Psychosocial Support as a Core Function and (4) Transparent Processes and Feedback. Transparent processes, clear communication of consequences and accessible psychosocial support are key to increasing trust and reporting willingness. Faculties should therefore ensure low-threshold access to advisory services, actively communicate reporting pathways, raise awareness and systematically monitor the effectiveness of institutional responses. Future research should evaluate whether implementing and strengthening these measures improves student satisfaction, institutional trust and ultimately contributes to reducing sexual harassment in medical education.

**STRENGTHS AND LIMITATIONS OF THIS STUDY**

- This study includes one of the largest national samples of medical students on sexual harassment in medical education in Germany and integrates three complementary data sources.
- The multi-method design, integrating three quantitative data sources, enabled a multi-level analysis of reporting structures, perceived barriers and institutional responses, informing practical recommendations for medical schools.
- The study was co-led by medical students, which strengthened the relevance and interpretation of findings, while reflexivity was supported through supervision by a senior clinical researcher.
- The student survey was based on voluntary participation, which may introduce self-selection bias, and the cross-sectional design does not permit causal inference.
- Findings may not directly generalise to countries with different higher education systems.

## Introduction

 Sexual harassment represents a significant problem in academic medicine, a field characterised by pronounced hierarchical structures.^1–3^ Medical students may be particularly vulnerable because they depend on supervisors for teaching, assessments and career progression. Internationally, a systematic review and meta-analysis of 51 studies reported that 33.1% of medical students and 36.2% of residents experienced sexual harassment during training, with no evidence of declining prevalence over time.^4^ Similar findings have been reported from medical schools in Switzerland, the UK, Canada and the USA, with prevalence estimates ranging from approximately 20% to nearly 60% depending on the study population and methodology.^5–10^ Studies from German-speaking countries also report high prevalence rates, particularly in clinical training environments.^3 5 11–13^ In our 2024 nationwide study, 44.2% of German medical students reported personal experiences of sexual harassment during their studies.^14^ Despite this high prevalence, formal reporting remains uncommon, as demonstrated in studies from the UK and Germany.^6 14^

Hierarchical dependency, high performance pressure and competitive institutional cultures can facilitate boundary violations,^15–17^ particularly in medical settings.^2^ Affected individuals frequently report psychological distress, loss of trust in institutions and impaired professional development.^3 5 18–20^ At the organisational level, sexual harassment is associated with organisational withdrawal and reduced institutional commitment.^21^

Institutions therefore carry a particular responsibility to provide preventive measures and effective support structures. These include transparent reporting pathways, trained contact persons and binding codes of conduct.^15 22–27^ Prior research suggests that the presence of such structures can not only have preventive effects but also increase the likelihood that affected individuals report incidents.^26 28 29^ In Germany, universities are legally required under the General Equal Treatment Act (*Allgemeines Gleichbehandlungsgesetz* (AGG)) and state higher education regulations to implement measures to protect against sexual harassment and discrimination.

Despite the growing body of evidence on the prevalence of sexual harassment, little is known about the institutional reporting and support structures available to medical students in Germany. Low reporting rates may reflect not only individual barriers but also differences in the availability, visibility and accessibility of institutional reporting structures. Previous international and German studies have focused primarily on prevalence and individual barriers to reporting. However, no nationwide study has systematically assessed institutional prevention frameworks, reporting pathways and support services across German medical faculties. Consequently, it remains unclear whether differences in institutional policies and support services are associated with formal reporting or students’ experiences with the reporting process.

The aim of this study was to investigate whether institutional reporting and support structures are associated with reporting behaviour and satisfaction among medical students in Germany. To this end, we integrated data from a nationwide online survey of medical students with publicly available information on reporting pathways and codes of conduct at 44 medical faculties, as well as survey data obtained from the respective deaneries.

The primary outcomes were whether affected students formally reported an incident and their satisfaction with the reporting process. Institutional reporting structures—including their availability, visibility, reporting pathways and support services—served as the main explanatory variables. Secondary outcomes included students’ awareness of institutional support structures, perceived accessibility of reporting pathways, the role of psychosocial support and perceived institutional consequences.

## Methods

The sampling frame comprised all German medical faculties offering a full undergraduate medical programme during the 2023/2024 academic year. These institutions constituted the target population for data collection. Data were collected sequentially from three sources. A nationwide online survey of medical students was conducted between June and September 2024. Additionally, a documentary analysis of publicly available institutional information was performed between April and May 2025. Finally, medical deaneries were contacted between May and July 2025 and invited to complete a standardised questionnaire. The institutional sampling frame comprised all German medical faculties represented in the nationwide student survey.

### Faculty survey

Between May and July 2025, we contacted all medical deaneries via email and invited them to complete a standardised questionnaire developed through an iterative process, using semi-structured multiple-choice items. The items were developed specifically for this study based on a review of the literature, drawing from the legal requirements of the General Equal Treatment Act (AGG),^30^ the recommendations of the Federal Anti-Discrimination Agency,^31^ expert recommendations for the design of reporting systems^24^ and the policy criteria proposed by Blumell *et al*.^26^ The survey assessed, among other aspects, the availability and accessibility of support services for medical students, as well as the scope of preventive and supportive measures addressing sexual harassment (online supplemental files 1; 2). Participation was voluntary and not anonymous. Items were reviewed by the research team for content validity before distribution.

### Review of publicly available information

In April and May 2025, we conducted a structured documentary analysis of publicly accessible information on the websites of all medical faculties. The website analysis followed a standardised search and extraction protocol developed a priori (full search and extraction protocol see online supplemental file 3). We identified documents that (a) explicitly addressed sexual or gender-based harassment/violence or (b) described reporting procedures applicable in such cases, always using the most recent available version. We collected data on equality and support services, codes of conduct and reporting procedures. We then carried out a comparative content analysis based on the criteria proposed by Blumell *et al*^26^ for institutional codes of conduct. We evaluated reporting pathways and the scope of available support services. The accessibility of reporting options was rated on a predefined scale (0=no dedicated service; 1=dedicated service via email/telephone only; 2=dedicated service also reachable via chat/messenger, including anonymous options; 3=online platform allowing anonymous reporting). We assessed the visibility of information on the websites using a second scale (0=no information available; 1=only accessible within embedded documents; 2=accessible via menu or search function; 3=prominently displayed, eg, on the homepage). Data collection was performed by a trained researcher and independently cross-validated by a second reviewer for a subset of cases. Websites on which no relevant information was found underwent a full secondary review. Any remaining discrepancies were discussed within the research team and resolved through consensus.

### Survey of medical students

A third dataset was derived from a nationwide anonymous online survey of medical students conducted between June and September 2024.

The survey instrument was developed iteratively based on previously published questionnaires assessing sexual harassment in medical education, particularly the instrument developed by Schoenefeld *et al*,^11^ and adapted to the German medical education context. Prior to nationwide administration, the questionnaire underwent pilot testing with medical students to assess comprehensibility, relevance and completeness. The survey comprised demographic questions and eight thematic domains; demographic variables included age, gender, study semester and university. Students were classified as being in the pre-clinical phase (semesters 1–4, prior to the First State Examination), the clinical phase (semester 5–10, prior to the Second State Examination) or the final year (prior to the Third State Examination). Prior non-medical vocational experience was defined as completion of a vocational training programme or university degree outside medicine before enrolment in medical school. To ensure a common understanding of the topic, the questionnaire was preceded by a definition of sexual harassment based on the German General Equal Treatment Act (AGG),^30^ illustrated with concrete examples. Participants were then asked whether they had personally experienced sexual harassment during their medical studies (yes/no). Those reporting personal experiences completed additional questions on the type, location, perpetrators, frequency, reporting behaviour, psychological burden, consequences and reporting behaviour, use of and satisfaction with support services, using multiple-choice and Likert-type response formats. The study design, sampling strategy and questionnaire development have been described in detail in a previous manuscript.^14^

For the present study, analyses focused on students’ use of institutional reporting and support structures, satisfaction with these services and subjective psychological burden following sexual harassment. Burden was rated on a numerical rating scale from 0 (not at all) to 10 (very severe); perceived consequences and satisfaction were assessed using five-point Likert scales. Reasons for not reporting were categorised into five domains (structural/process-related, emotional, career-related consequences, uncertainty about classification and anticipated lack of consequences) based on existing literature and an accompanying qualitative interview study^32^ and assessed as multiple-response options.^14^

### Data analysis

Survey data from students were linked to institutional characteristics and deanery responses based on university affiliation. The sample was described descriptively. For analyses at the faculty level, only faculties with at least 50 completed student survey responses were included. This threshold was chosen to ensure that faculty-level estimates were based on a sufficient number of respondents to provide stable descriptive estimates, facilitate more robust comparisons with institutional characteristics and to reduce the influence of random variation resulting from very small sample sizes.

As key variables were not normally distributed, medians and IQRs are reported. To examine associations between structural factors and (a) reporting behaviour, (b) satisfaction with the reporting process and (c) willingness to report again, we conducted multiple regression analyses: a logistic regression predicting the likelihood of reporting sexual harassment, and linear regressions predicting satisfaction and willingness to report again. Categorical predictors (eg, prior degree, stage of study) dummy coded using predefined reference categories, dichotomous indicators (eg, presence of preventive measures) and numerically coded Likert-scale variables (student ratings on five-point scales) were included. Statistical significance was defined as p<0.05. Analyses were performed using Python 3.11.8 in Visual Studio Code 1.87.1. Finally, we compared data from the student survey with the accessibility of counselling services as reported by the deaneries and the visibility of information derived from our website analysis in a heatmap for pattern analysis.

## Results

A total of 42 medical faculties were identified in Germany, including 39 public and 3 private or partner institutions.

### Results of the faculty survey

34 of the 42 faculties completed the questionnaire (response rate: 81.0%). The majority of deaneries reported providing a dedicated counselling service for medical students. Approximately half of these services have been in place for more than 5 years, while 13 were established within the past 5 years and two were implemented in the year preceding the survey. Most faculties indicated that anonymous reporting is generally possible; however, only 29.4% reported offering a corresponding online form. Support services primarily focused on coordination with other institutional offices, while preventive measures mainly emphasised empowerment and awareness-raising (table 1).

**Table 1:** Deanery responses regarding counselling services for sexual harassment

Item	Answers
Dedicated counselling services for medical students	Yes: 88.2% (30), of which:* University: 69.7% (23) Medical faculty: 61.8% (21) University hospital: 33.3% (11) No: 11.8% (4)
Implementation of the counselling service	< 1 year: 5.9% (2) 1–5 years: 38.2% (13) >5 years: 44.1% (15) No information: 11.8% (4)
Formal evaluation of the counselling service ever conducted	Yes: 47.1% (16) Unsure: 32.4% (11) No: 26.5% (9)
Accessibility*	Email: 94.1% (32) In person: 91.2% (31) Phone: 91.2% (31) Online form: 29.4% (10) Online chat: 8.8% (3)
	Anonymous reporting possible: 64.7% (22)
Support services offered*	Coordination with other offices: 88.2% (30) Psychological counselling: 52.9% (18) Legal advice: 50.0% (17) Initiation of disciplinary procedures: 29.4% (10)
Preventive measures*	Training for students: 50.0% (17) Training for faculty: 55.9% (19) Content integrated into leadership training: 50.0% (17) Awareness-raising/empowerment activities: 85.3% (29)

* Multiple responses possible.

### Results of the analysis of publicly available information

Policy documents or codes of conduct were available at nearly all faculties (41/42; 97.6%). References to sexual harassment were found in 41 and to discrimination in 40 cases. The content of these documents largely met established recommendations for institutional guidelines. All documents included a definition of sexual harassment; most explicitly referred to hierarchical dependencies (73.2%) and stated that harassment cannot be provoked (78.0%). A zero-tolerance policy was declared in 85.7% of faculties’ publicly available documents.

**Figure 1:**
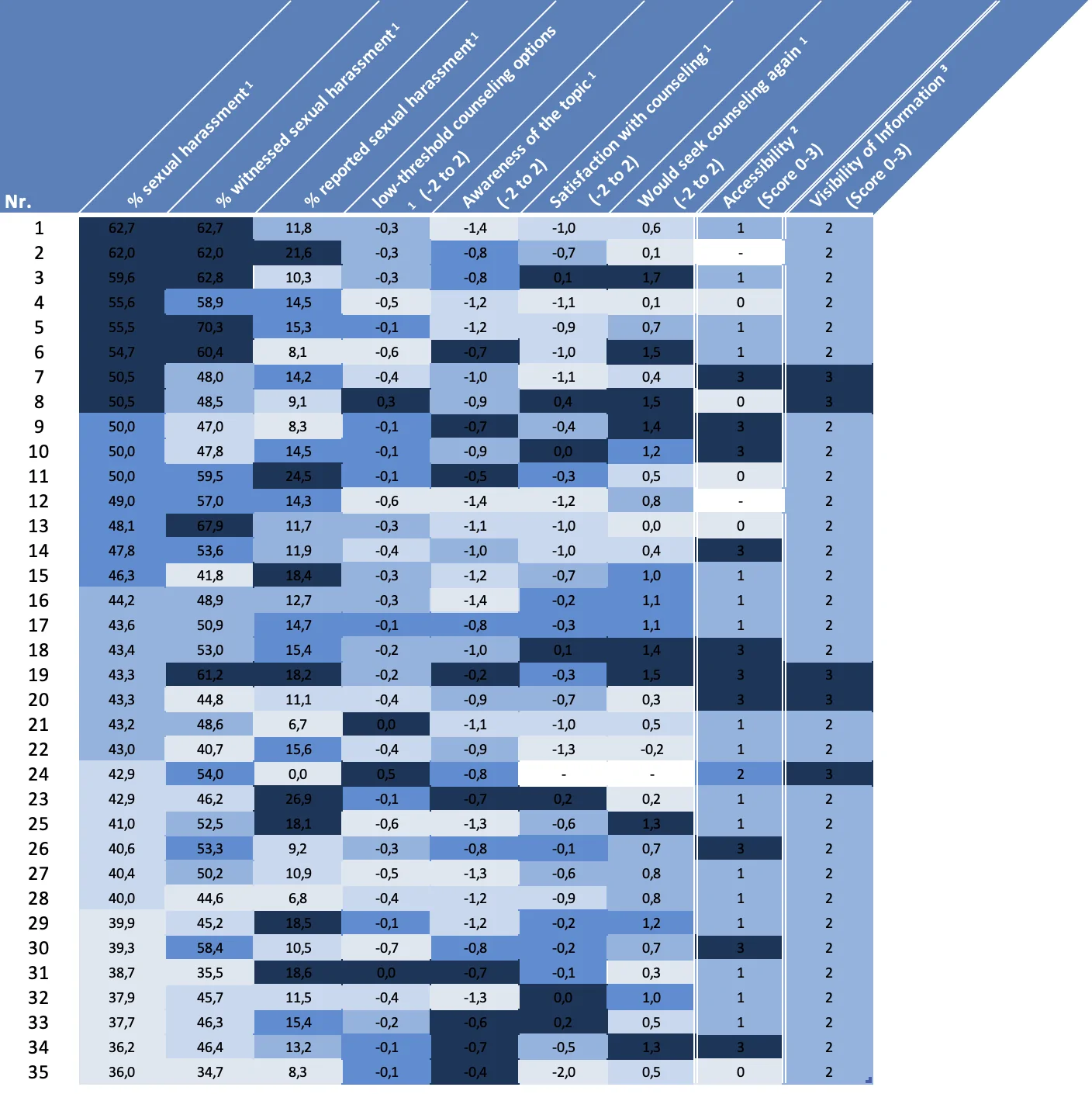
Overview of participating faculties. ¹ Student survey. ² Faculty deans’ survey. ³ Publicly available online information. Only faculties with≥50 respondents in the student survey were included. The accessibility score captures the ease of access and anonymity of reporting options: (0) no dedicated counselling service, only a general referral (non-anonymous), (1) dedicated reporting office, reachable only via email/phone (non-anonymous), (2) dedicated office also reachable via chat/messenger (anonymous option available) and (3) online platform enabling (anonymous) reporting. The information visibility score describes how easily information about the reporting office can be found on the website: (0) no information available, (1) only hidden within documents, (2) accessible via the main menu or search function and (3) prominently displayed (eg, on the homepage).

Counselling and reporting offices could be identified via faculty websites in 37 cases (88.1%). These offices more frequently reported offering psychological support (83.3%) than indicated by the deaneries. Legal counselling was advertised at 19 sites (45.2%). Anonymous online reporting platforms were available at four institutions (9.5%). Information was generally accessible via search functions or website menus (81.0%) but was rarely prominently displayed (11.9%) (figure 1). A description of the reporting or case-handling process was available in 89.2% of cases, while concrete timelines for response or case resolution were provided in only 18.9%; a further 5.4% included vague references (‘promptly’). The majority of faculties explicitly stated guarantees of confidentiality (78.6%) and protection from retaliation for reporting individuals (80.1%) in the information provided on their websites.

### Student survey

A total of 5681 medical students from 44 faculties participated in the survey (77.8% female); complete datasets were available for 4158 respondents. 2392 students reported personal experiences of sexual harassment and 2805 reported having witnessed incidents. When considering only faculties with ≥50 participants (n=35), the proportion of affected students ranged from 36.0% to 62.7% (median 44.2%, IQR 9.9%) (figure 1).

**Figure 2:**
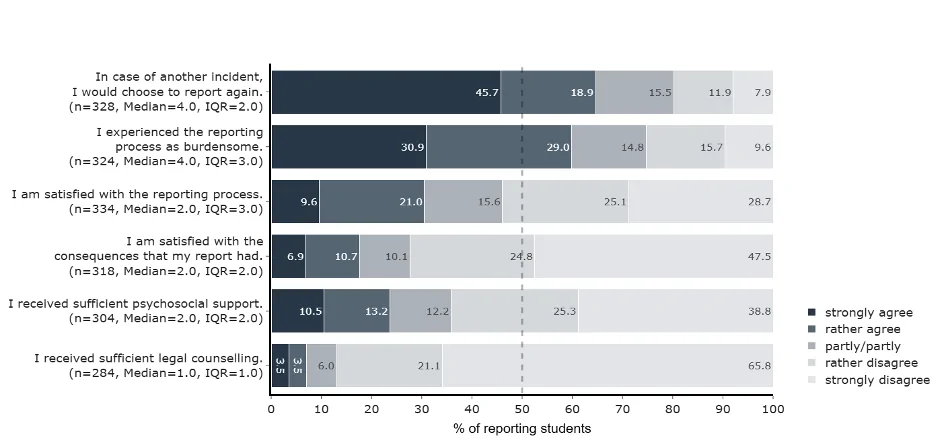
Satisfaction and perceived distress during the reporting process. Stacked bar chart illustrating participants’ ratings of satisfaction and perceived distress during the reporting process of sexual harassment. Only respondents who reported a witnessed or personally experienced incident are included. Percentages refer to responses on a five-point Likert scale (1=strongly disagree to 5=strongly agree). Below each statement, sample size (**n**), median and IQR of the numerical coding are provided.

**Figure 3:**
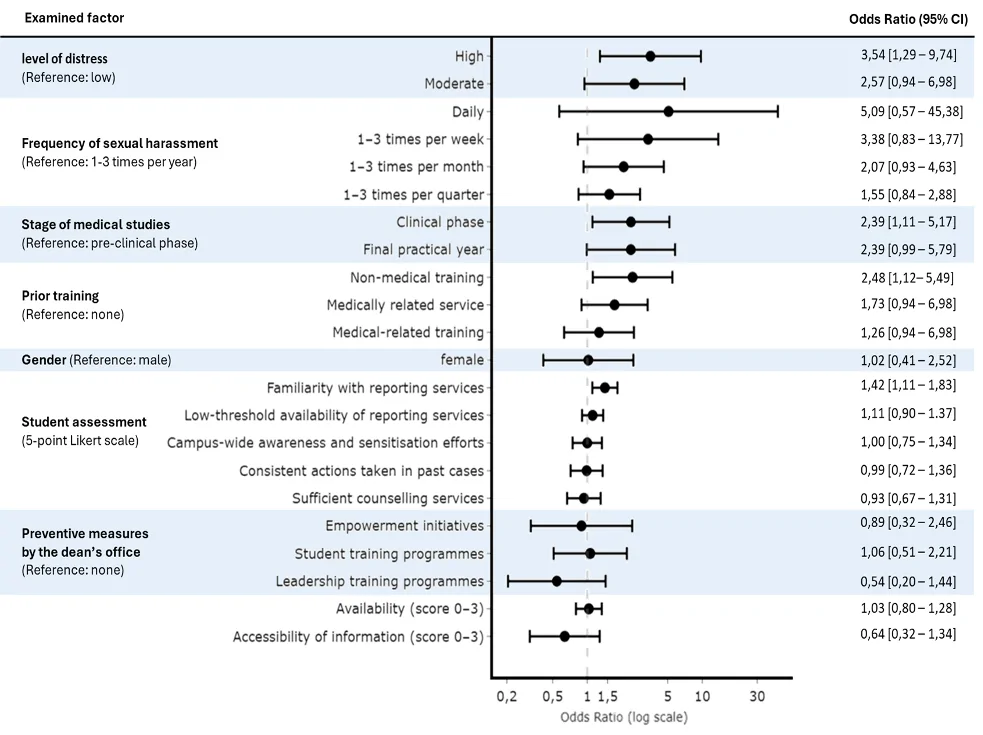
Regression analysis of students’ reporting behaviour. Forest plot illustrating the logistic regression analysis of medical students’ reporting behaviour in cases of sexual harassment. Displayed are ORs with 95% CIs; reference categories are indicated accordingly.

Only 12.7% of affected students reported having formally reported an incident, while 64.3% had not. Reporting rates did not differ significantly by gender and varied between faculties with a median of 12.0% (IQR 6.8%, n=35) (figure 1). Although many respondents described the reporting process as distressing, most indicated they would report again if faced with a similar situation (figure 2). The reporting process was evaluated similarly by male and female students. A significant proportion of those who had filed a report were dissatisfied with the reporting process and perceived consequences (figure 2). Overall, 48.6% of all respondents stated that they had not observed consistent institutional action against sexual harassment at their faculty, a proportion that rose to 81.6% among affected students (figure 3).

The reasons for not reporting incidents of sexual harassment were heterogeneous (n=2043). The most frequently cited reason was uncertainty about how to classify the incident (71.7%), followed by the expectation that reporting would have no consequences (61.8%). Other common reasons included unclear reporting procedures (48.5%), fear of negative personal repercussions (43.6%) and emotional distress (41.3%). The distribution of reasons did not differ substantially between genders.

**Figure 4:**
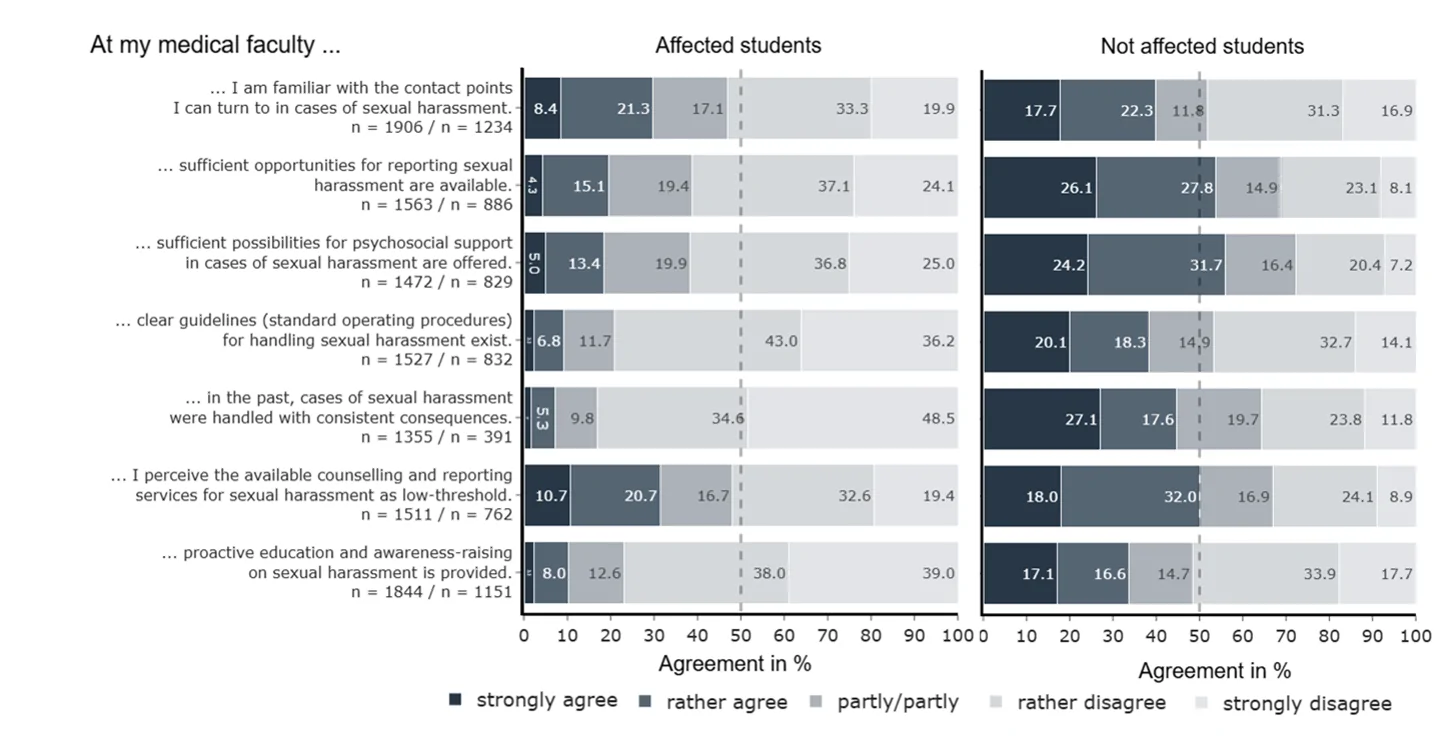
Perceptions of institutional response to sexual harassment. Stacked bar charts illustrating affected students’ perceptions of how sexual harassment is addressed at their medical faculty (left), compared with students who neither experienced nor witnessed harassment (right). Percentages represent response distributions on a five-point Likert scale (1=strongly disagree to 5=strongly agree).

Students without personal experience of sexual harassment rated existing institutional structures significantly more positively than those affected; the latter perceived reporting and support services as less adequate (figure 4).

### Factors influencing reporting behaviour, perceived distress and satisfaction

The logistic regression analysis (Pseudo-R² = 0.10; n=470) indicated that reporting behaviour was primarily associated with the level of distress experienced as a result of sexual harassment. Students who reported high distress were approximately 3.5 times more likely to file a report compared with those with lower distress levels (OR=3.48; 95% CI 1.67 to 7.27; p<0.001). Reporting was also more frequent among students with prior non-medical training (OR=2.6) and those in the clinical phase of study compared with pre-clinical students. There was a trend toward higher reporting with increasing frequency of harassment, although this did not reach statistical significance.

No significant associations were found for gender (OR=1.01; 95% CI 0.40 to 2.52; p=0.99) or for students’ perceptions of the accessibility of reporting pathways, institutional consistency in handling previous cases or adequacy of available support.

Likewise, the presence of faculty-level prevention measures or the accessibility of counselling and reporting services as reported by the deaneries showed no significant relationship with students’ willingness to report. In our analysis, no clear link emerged between perceived accessibility and the availability of online or anonymous reporting options. Overall, no consistent pattern of association was observed (figure 1).

The regression analysis of satisfaction with the reporting process revealed a strong positive association with the perceived consequences of institutional actions: with each step increase on the 5-point scale, satisfaction rose by an average of 0.49 points (p<0.001). Psychological support received during the process was also significantly associated with greater satisfaction (β=0.35; p<0.001), whereas legal support showed no meaningful effect (β=0.04; p=0.059). Preventive measures such as awareness or empowerment training as reported by the deaneries showed no significant relationship. Overall, the model explained a substantial proportion of the variance in satisfaction (Pseudo-R² = 0.75; n=201).

Similarly, the regression predicting willingness to report again showed that both perceived consequences (β=0.22; p=0.009) and psychological support (β=0.22; p=0.006) were positively associated with the intention to report again, whereas institutional characteristics of reporting offices were not.

## Discussion

The present study reveals a pronounced discrepancy between the high prevalence of sexual harassment and the very low rate of formal reporting among medical students. Although more than 40% of respondents reported personal experiences of sexual harassment, only 12.7% of those affected (304 of 2,392) formally reported at least one incident. These findings align with the well-documented international pattern of high prevalence yet persistently low reporting rates.^6 29 33 34^

In our regression analysis, reporting was primarily driven by the perceived psychological burden. The higher reporting odds among students with prior non-medical vocational experience and those in the clinical stage may reflect greater age, prior professional socialisation and familiarity with formal workplace procedures.

### Comparison of institutional structures with students’ perceptions

Under the German General Equal Treatment Act (AGG)^30^ and higher education regulations, universities are legally required to implement procedures that protect against discrimination and sexual harassment. All faculties included in this study met this formal requirement and reported offering corresponding counselling services. However, there were marked differences in the design, visibility and accessibility of these structures.

Only a minority of faculties currently provide online or chat-based reporting options. However, our analysis did not reveal a significant association between students’ reporting behaviour and the availability of online platforms. Similarly, no link was found between perceived accessibility and the objectively measured reachability of services. This suggests that perceived accessibility depends less on technical features and more on visibility, trust and perceived approachability.

Our findings further indicate that affected students perceive psychosocial support as substantially less available than reported by the faculties themselves. This discrepancy suggests that existing offices are often perceived by students primarily as formal reporting bodies rather than supportive counselling services. Furthermore, the positive association between psychological support and student satisfaction emphasises the critical role of accessible psychosocial services. Psychosocial support should therefore be more clearly established as a core function of these structures.

A similar gap was observed in the area of prevention: while 29 out of 34 faculties reported implementing awareness and empowerment initiatives, only 31.1% of students confirmed proactive education on sexual harassment at their institution. Among affected students, this rate dropped to 11.4%. This indicates that current measures are not sufficiently reaching their target groups.

## Barriers to reporting

In our survey, the most cited reasons for not reporting sexual harassment were uncertainty about how to interpret the incident and the expectation that reporting would not lead to any consequences. Among affected students, 48.5% stated that they did not report an incident due to unclear or unknown reporting pathways. Although most counselling services could be located online in our analysis, students often appeared unaware of their existence or did not perceive them as relevant or usable in concrete situations. Faculties should therefore work proactively to make existing structures visible - for example, by presenting them in teaching sessions and orientation programmes, integrating them into digital learning platforms and promoting them through posters, flyers or awareness campaigns.

Among those who had used institutional reporting mechanisms, satisfaction with the process and willingness to report again were strongly associated with the perceived effectiveness of institutional responses and the experience of psychosocial support. These findings align with international research showing that both students and medical staff often feel unsure whether an incident is ‘serious enough’ or whether a report would have any effect.^35 36^ In learning environments, hierarchies and fear of negative repercussions frequently deter individuals from reporting.^28^ Commonly cited barriers include fear of retaliation,^6 37^ difficulties in judging the incident, feelings of shame and lack of trust in institutional responses.^35 38 39^ Systemic factors also contribute, such as unclear or time-consuming reporting procedures.^36^

In hierarchical systems, the responsibility for addressing harassment is often individualised, while structural causes remain unacknowledged.^40 41^ However, sexual harassment occurs not only in individual interactions but within a broader professional culture.^25 42^ Particularly in highly hierarchical and performance-driven environments, cultural factors can contribute to the normalisation of boundary violations, placing students and early-career professionals in a tension between social expectations and professional dependency.^16 28 40 43 44^

Previous studies have shown that the decision to report incidents is strongly influenced by individual attitudes, social norms within the environment and the perceived effectiveness of institutional structures.^6 28 29 33 35 37–39 45 46^ A key factor in this process is trust - the belief that an incident will be taken seriously and handled appropriately. Clear procedures, visible consequences and trustworthy points of contact can significantly increase the likelihood of reporting, whereas unclear or inconsistent institutional responses have the opposite effect.^28^ Drawing on the *Theory of Planned Behaviour*, prior research has demonstrated that the expectation of real consequences and the perception of personal safety and reputational protection are major predictors of reporting behaviour.^45^ Our regression analyses support these findings, as perceived consequences of institutional action were closely linked to students’ satisfaction with the reporting process. This highlights the importance of transparent communication and structured feedback mechanisms for affected individuals.

The goal of every faculty should be that students both know these services and perceive them as trustworthy points of contact.

### Implications for policymakers

According to the Federal Anti-Discrimination Agency of Germany, an effective complaints procedure is characterised by its adaptation to the local context and the clear definition of responsibilities and processes.^22 27^ Furthermore, regular monitoring and transparent feedback on reports and measures are recommended to ensure procedural quality and strengthen trust among those affected.^24^

Additional key recommendations for effective reporting systems include (1) a clearly communicated zero-tolerance culture, (2) appropriate and proportionate measures in response to reports, (3) protection against retaliation, (4) low-threshold and accessible reporting channels and (5) structured institutional monitoring.^24^ These principles are consistent with our findings: transparency, visibility and perceived effectiveness—rather than the mere existence of structures—are crucial for students’ willingness to report and their satisfaction with the process.

Based on our data, four practical recommendations for medical faculties can be derived:

#### Awareness and competence-building

Embed awareness and competence training as a continuous element of medical education and clinical practice. All members of the academic community should be equipped to recognise, address and prevent harassment.

#### Visibility and active communication

Present reporting and counselling services proactively in orientation sessions, curricula and digital platforms, and promote their clear visibility across campus and clinical settings.

#### Psychosocial support as a core function

Establish counselling services as confidential, supportive contact points focused on guidance and relief rather than formality or sanctions.

#### Transparent processes and feedback

Define procedures and responsibilities clearly and provide regular feedback to those affected on the progress and outcomes of their reports.

### Strengths and limitations

A strength of this study lies in the combination of student perspectives, publicly available institutional information and data provided by student deaneries, allowing for a comprehensive overview of reporting and support structures at German medical faculties.

As a cross-sectional study, the analysis does not permit causal inferences. Voluntary participation entails the risk of self-selection bias; however, the observed prevalence and distress patterns are consistent with findings from previous international research. The analysis of reporting and counselling structures was based on publicly available information, meaning that services hosted within internal networks (eg, intranets) may not have been captured. Furthermore, ordinal Likert-scale data were numerically coded, which may limit the interpretability of specific effect sizes. Since the student and deanery surveys were conducted at different time points, subsequent structural changes could not be accounted for. Therefore, the findings should be interpreted as reflecting structural patterns and perception tendencies, rather than as causal evidence of specific relationships.

A denominator-based response rate could not be calculated because the survey was distributed through multiple open recruitment channels. The study included responses from 5681 medical students, representing approximately 6% of all medical students enrolled in Germany. As with all voluntary online surveys, selection bias cannot be excluded, and students with personal experiences of sexual harassment may have been more likely to participate. Women were over-represented (77.8%) relative to the national average of medical students (64.6% in 2023), which may bias prevalence estimates. Although the large nationwide sample from 44 medical faculties provides broad insight into students’ experiences across German medical education, findings should be interpreted with this limitation in mind. By contrast, the deanery survey achieved a high response rate (81.0%, 34 of 42 faculties), which strengthens the institution-level findings.

### Future research

Future research should explore how reporting and support structures are actually experienced and used by students, for example through qualitative investigations involving affected individuals, counselling services and teaching staff. Likewise, the evaluation of existing awareness and support programmes is needed to identify and further develop effective models.

At the institutional level, systematic monitoring appears crucial: regular collection and transparent communication of reports, procedures and outcomes can help build trust and contribute to quality improvement. Furthermore, awareness and prevention efforts should not be treated as one-time interventions but rather be integrated continuously across teaching and clinical practice. Future studies should evaluate longitudinally whether improved visibility, psychosocial support and transparent feedback mechanisms increase reporting rates and satisfaction among affected students.

## Conclusion

Reporting and counselling structures exist at most medical faculties, yet affected students often do not perceive them as visible, accessible or effective. Our findings indicate that it is not the mere existence of these services that matters, but rather their transparency, approachability and the perceived consequences of institutional responses. Confidential psychosocial support and clearly communicated, comprehensible follow-up measures after a report are important.

For faculties, this implies a clear mandate for action: awareness and education on sexual harassment must be firmly integrated into medical curricula, teaching and clinical practice, supported by an institution-wide zero-tolerance culture. Counselling services should be low-threshold and actively promoted, and reporting procedures must be transparent and reliable. Establishing such structural and cultural foundations can help not only to address but to prevent sexual harassment sustainably, fostering a safe, respectful and inclusive environment for medical students.

## Supplementary material

10.1136/bmjopen-2025-114374 online supplemental file 1

10.1136/bmjopen-2025-114374 online supplemental file 2

10.1136/bmjopen-2025-114374 online supplemental file 3

## Data Availability

Data are available upon reasonable request.
