# Supplementary material for: Sexual harassment at medical schools: barriers to reporting and strategies for improvement - a nationwide, cross-sectional, multi-method study from Germany
Source: BMJ Open. 2026 Aug 21;16(8):e114374. doi: 10.1136/bmjopen-2025-114374 (PMC13504847; doi:10.1136/bmjopen-2025-114374)
Supplement: Supplementary file 1 [file bmjopen-16-8-s001.pdf]

Umfrage zu Meldestrukturen und Beratungsstellen für Diskriminierung und sexuelle Belästigung im Medizinstudium

Sehr geehrte Damen und Herren,

im Rahmen eines Projekts zur Erhebung und Verbesserung von Schutz- und Unterstützungsstrukturen für Medizinstudierende in Deutschland möchten wir uns einen Überblick über bestehende Meldestellen und Beratungsangebote an den Fakultäten verschaffen. Besonders interessiert uns, wie Medizinstudierende bei Diskriminierung oder sexueller Belästigung unterstützt werden – auch in Kontexten außerhalb der Universitätsgebäude, z. B. während Famulaturen oder Praktika im klinischen Alltag.

○ Hiermit bestätige Ich, dass Ich für die Medizinische Fakultät Standort Aussagen zu den Meldestrukturen nach bestem Wissen und Gewissen beantworte.

1. Zuständigkeit und Einrichtung

1.1. Alle Hochschulen in Deutschland bieten grundsätzlich Meldestellen für Studierende bei Diskriminierung und sexueller Belästigung an. Jedoch zeigen sich im Medizinstudium strukturelle Lücken – etwa durch kliniknahe Ausbildungsabschnitte außerhalb universitärer Räume.

Gibt es an Ihrer Fakultät eine Meldestelle, die für die Belange von Medizinstudierenden bei Diskriminierung oder sexueller Belästigung ausgerichtet ist?

☐ Ja ☐ Nein ☐ Weiß ich nicht

1.2. Von welcher Institution wird die Meldestelle oder Beratungsstelle betrieben an die sich Medizinstudierende vorrangig wenden sollen?

☐ Universität ☐ Medizinische Fakultät

☐ Universitätsklinikum Sonstiges: ______________________

1.3. Welchen beruflichen Hintergrund haben die Mitarbeitenden der Meldestelle überwiegend?

____

1.4. Wann wurde die Meldestelle eingerichtet?

____

1.5. Wird Nutzung und Qualität der Meldestelle regelmäßig evaluiert?

☐ Ja ☐ Nein ☐ Weiß ich nicht

Wenn ja, wie? ____

2. Erreichbarkeit und Anonymität

2.1. Welche Meldewege stehen zur Verfügung?

☐ Telefonisch ☐ Online Chat

☐ Online Formular ☐ Persönlich

☐ E-Mail Sonstiges: ____________________

2.2. Ist eine anonyme Meldung möglich?

☐ Ja ☐ Nein ☐ Weiß ich nicht

3. Richtlinien und Beratung

3.1. Existiert eine Richtlinie oder ein Code of Conduct zur sexuellen Belästigung, herausgegeben von:

☐ Universität ☐ Medizinische Fakultät

☐ Universitätsklinikum Sonstiges: ____________________

3.2. Optional: Wo ist diese Richtlinie zugänglich?

Link: ____

3.3. Welche Unterstützungsangebote bietet die Beratungsstelle?

☐ Rechtliche Beratung ☐ Psychologische Beratung

☐ Einleitung von Disziplinarverfahren ☐ Koordination mit anderen Stellen

Sonstiges: ____________________

4. Prävention und Organisationsentwicklung

4.1. Gibt es Maßnahmen in folgenden Bereichen für Studierende?

Organisations- und Personalentwicklung (z.B. Verpflichtende Fortbildungen für Personalverantwortliche, Angebote für Fort- und Weiterbildung) ☐ Ja ☐ Nein ☐ Weiß ich nicht

Infrastruktur (z.B. Schaffen räumlicher Bedingungen zur Vermeidung von Gefahrensituationen) ☐ Ja ☐ Nein ☐ Weiß ich nicht

Sensibilisierung und Empowerment (z.B. Plakat-Kampagnen, Workshops) ☐ Ja ☐ Nein ☐ Weiß ich nicht

4.2. Gibt es Maßnahmen in folgenden Bereichen für Lehrende?

Organisations- und Personalentwicklung ☐ Ja ☐ Nein ☐ Weiß ich nicht

Infrastruktur ☐ Ja ☐ Nein ☐ Weiß ich nicht

Sensibilisierung und Empowerment ☐ Ja ☐ Nein ☐ Weiß ich nicht

5. Kontakt und Rückfragen

Für eventuelle Rückfragen, weiterführende Gespräche oder Interesse an den Ergebnissen Ihrer Fakultät zur Umfrage unter den Studierenden bezüglich sexueller Belästigung aus dem Jahr 2024, nennen Sie uns bitte eine Ansprechperson:

Name: ______________________ Email: ______________________
