# Supplementary material for: Sexual harassment at medical schools: barriers to reporting and strategies for improvement - a nationwide, cross-sectional, multi-method study from Germany
Source: BMJ Open. 2026 Aug 21;16(8):e114374. doi: 10.1136/bmjopen-2025-114374 (PMC13504847; doi:10.1136/bmjopen-2025-114374)
Supplement: Supplementary file 2 [file bmjopen-16-8-s002.pdf]

Survey on reporting structures and counseling centers for discrimination and sexual harassment in medical studies

Dear Sir or Madam,

As part of a project to survey and improve protection and support structures for medical students in Germany, we would like to gain an overview of existing reporting centers and counseling services at the faculties. We are particularly interested in how medical students are supported in cases of discrimination or sexual harassment – including in contexts outside the university buildings, e.g. during clinical placements or internships in everyday clinical practice.

- ☐ I hereby confirm that I am answering questions about the reporting structures for the Faculty of Medicine to the best of my knowledge and that the data may be used anonymously for scientific evaluation and publication.

1. Responsibility and institution
1.1. All universities in Germany generally offer reporting offices for students in cases of discrimination and sexual harassment. However, there are structural gaps in medical studies – for example, due to clinic-based training periods outside university premises.

Does your faculty have a reporting office that deals with issues of discrimination or sexual harassment affecting medical students?

- ☐ Yes
- ☐ No
- ☐ I don't know

1.2. Which institution operates the reporting office or counseling center that medical students should primarily contact?

- ☐ University
- ☐ Medical school
- ☐ University hospital
- ☐ Other: *Free text*

1.3. How are the employees of the reporting office in question trained?
Free text

1.4. When was the reporting office established?

- [ ] <1 year
- [ ] 1-5 years
- [ ] >5 years
- [ ] I don't know

1.5. Are the use and quality of the reporting office evaluated regularly?

- [ ] Yes
- [ ] No
- [ ] I don't know

If yes, how?
Free text

2. Accessibility and anonymity

2.1. What reporting channels are available?

- [ ] By telephone
- [ ] Online chat
- [ ] Online form
- [ ] In person
- [ ] Email
- [ ] Other: *Free text*

2.2. Is anonymous reporting possible?

- [ ] Yes
- [ ] No
- [ ] I don't know

3. Guidelines and counseling

3.1. Is there a guideline or code of conduct on sexual harassment issued by:

- [ ] University
- [ ] Medical school
- [ ] University hospital
- [ ] Other: *Free text*

3.2. Optional: Where can this guideline be accessed?
Link: *Free text*

3.3. What support services does the counseling center offer?

- [ ] Legal counseling
- [ ] Psychological counseling
- [ ] Initiation of disciplinary proceedings
- [ ] Coordination with other agencies
- [ ] Other: *Free text*

4. Prevention and organizational development

4.1. Are the following measures in place?	(Yes | No | I don't know)		
Training opportunities for students	▢	▢	▢
Training opportunities for lecturers	▢	▢	▢
Issue addressed in management training	▢	▢	▢
Awareness raising and empowerment (e.g., poster campaigns, flyers, information events)	▢	▢	▢

Other (optional): *Free text*

5. Contact and queries

If you have any questions, would like to discuss the matter further, or are interested in the results of your faculty's 2024 student survey on sexual harassment, please provide us with the details of a contact person:

Name:

Email:
