# Supplementary material for: Sexual harassment at medical schools: barriers to reporting and strategies for improvement - a nationwide, cross-sectional, multi-method study from Germany
Source: BMJ Open. 2026 Aug 21;16(8):e114374. doi: 10.1136/bmjopen-2025-114374 (PMC13504847; doi:10.1136/bmjopen-2025-114374)
Supplement: Supplementary file 3 [file bmjopen-2025-114374.reviewer_comments.pdf]

PEER REVIEW HISTORY

BMJ Open publishes all reviews undertaken for accepted manuscripts. Reviewers are asked to complete a checklist review form and are provided with free text boxes to elaborate on their assessment. These free text comments are reproduced below.

ARTICLE DETAILS

Title (Provisional)

Sexual Harassment at Medical Schools: Barriers to Reporting and Strategies for Improvement. A Nationwide, Cross-Sectional, Multi-Method Study from Germany

Authors

Vogt, Maximilian; Förstel, Michelle; Drossard, Sabine

VERSION 1 - REVIEW

**Reviewer**	**1**
**Name**	**Dehghan, Mahlagha**
**Affiliation**	**Kerman University of Medical Sciences**
**Date**	**05-Jan-2026**
**COI**	**None**

Dear authors,

Abstract

1- the "Cross-sectional mixed-method study" does not seem correct. Please clearly explain which type of mixed-method study it is.

2- in the method the quantitative and qualitative sections are not clear.

3- in the results it is not clear what was the qualitative results!

4- "We propose four additional strategies for medical faculties: (1) Awareness and Competence-Building, (2) Visibility and Active Communication, (3) Psychosocial Support as a Core Function and (4) Transparent Processes and Feedback." Seems to be results not conclusion.

Introduction

1- evidence for the similar articles in other countries are not well reported.

2- the gap is not well justified.

3- please clearly state the objectives of the study.

Methods

1- please clearly explain about the study design. It is not clear the study has a mixed method design or only the authors used triangulations for gathering data.

2- please report the method section according to relevant guidelines.

3- “All German medical faculties offering a full undergraduate medical program in the 2023/24 academic year were included.” “Between May and July 2025, we contacted all medical deaneries via email and invited them to complete” why the sampling period is different?

4- please explain about the validity and reliability of all tools used for data collection.

5- “For analyses at the faculty level, only faculties with at least 50 completed student survey responses were included.” Why?

6- how about the ethical considerations?

Results

1- in table one some percentages are more than 100%!

2- what does it mean by “Evaluation” in table 1.

3- “personal experiences of sexual harassment” how were assessed?

Best regards,

**Reviewer**	**2**
**Name**	**CARTA, Angela**
**Affiliation**	**University of Verona, Diagnostics and Public Health**
**Date**	**21-Jun-2026**
**COI**	**None**

The manuscript addresses the important issue of sexual harassment, focusing on medical students. The authors have adopted a mixed-methods approach, conducting a national survey to identify the most significant barriers and provide guidance for future prevention strategies.

There are no particular concerns regarding the article's content or scientific validity, but it would be best to consider certain suggestions before publication.

On page 3, lines 47–57, it would be advisable to add a concluding sentence on future research.

On page 5, lines 6–15: To give the reader an idea of the known extent of the phenomenon, it might be useful to add data on prevalence. Lines 35–40: The aim of the study is clearer in the abstract than here. It would be preferable to rephrase and explain the aim more clearly, bearing in mind that the description of the methodology referred to is, in any case, set out in

the methods section. In these lines, the primary objectives and, where applicable, the secondary objectives of your study should be stated more clearly. Lines 52–54: It would be advisable to include a brief explanation of the ‘iterative process’ used to develop the questionnaire, together with references to the theoretical sources that guided its development, where applicable.

On page 6, line 31: the reference cited in the text as a source of information on the questionnaire should be revised. Currently, the manuscript cannot be considered a ‘previous manuscript’, as it is a manuscript under review that has not yet been accepted for publication. It is therefore advisable to provide a brief general description of the questionnaire's main dimensions to facilitate a better understanding of the methodology adopted.

On page 8, lines 29–34: What is the response rate among medical students? Although 5,691 students constitute a large population, it is important to assess how representative this sample is. Response rates are often low in online surveys on this topic; therefore, it is important to discuss the representativeness and implications of these data.

On page 9, lines 15–19: a brief explanation (either here or in the ‘Methods’ section) is required of the categorisation of students into ‘non-medical background’, ‘students in clinical stages’ and ‘pre-clinical students’, and the result should be briefly discussed in the ‘Discussion’ section.

On page 12, lines 23–26, a brief comment should be added regarding the data's representativeness. The response rate reported by the medical schools is high and should be considered a strength; on the other hand, as noted above, the response rate among medical students warrants brief consideration in light of comparisons with the literature.

VERSION 1 - AUTHOR RESPONSE

Reviewer 1

Dr. Mahlagha Dehghan, Kerman University of Medical Sciences

Comments to the Author:
Dear authors,
Abstract
1- the “Cross-sectional mixed-method study” does not seem correct. Please clearly explain which type of mixed-method study it is.
+ 2- in the method the quantitative and qualitative sections are not clear.
+ 3- in the results it is not clear what was the qualitative results!

We agree. As no qualitative data are reported, the design is now described as a multi-method study integrating three quantitative data sources, and the abstract Design and Methods statements have been rewritten to name these sources explicitly.

4- “We propose four additional strategies for medical faculties: (1) Awareness and Competence-Building, (2) Visibility and Active Communication, (3) Psychosocial Support as a Core Function and (4) Transparent Processes and Feedback.” Seems to be results not conclusion.

We agree these are derived recommendations rather than empirical findings. In the abstract Conclusion we now frame them explicitly as recommendations rather than results, and in the main text they appear under “Implications for Policymakers” as recommendations derived from the data.

<u>**Introduction**</u>

1- evidence for the similar articles in other countries are not well reported.

We have expanded the Introduction with international evidence. It now cites the systematic review and meta-analysis by Fnais et al. (Acad Med 2014) and refers to studies from the UK US, Switzerland and Canasa demonstrating the same pattern of high prevalence and low formal reporting.

2- the gap is not well justified.

We have sharpened the gap statement in the final Introduction paragraph. It now states explicitly that, while previous international and German studies document prevalence and individual barriers to reporting, no national study to date has linked institutional reporting and support structures — assessed from both institutional self-reports and publicly available information — to students’ actual reporting behaviour and satisfaction. This is the gap the present study addresses.

3- please clearly state the objectives of the study.

The objectives are now stated explicitly at the end of the Introduction, including the primary and secondary outcomes.

<u>**Methods**</u>

1- please clearly explain about the study design. It is not clear the study has a mixed method design or only the authors used triangulations for gathering data.

Clarified. We describe the design as a cross-sectional multi-method study and state that the three quantitative datasets were linked at the faculty level and integrated analytically (regression analyses and a cross-source heatmap), which goes beyond simple triangulation of independent data collection.

2- please report the method section according to relevant guidelines.

We now report the study in accordance with STROBE for cross-sectional studies. The completed STROBE checklist, indicating page and line numbers, is submitted with the revised manuscript.

3- “All German medical faculties offering a full undergraduate medical program in the 2023/24 academic year were included.” “Between May and July 2025, we contacted all medical deaneries via email and invited them to complete” why the sampling period is different?

Thank you for pointing this out. The 2023/24 academic year defines the sampling frame (i.e., which faculties were eligible), not the data-collection period. The three data sources were collected sequentially: the student survey between June and September 2024, the documentary website analysis in April–May 2025, and the deanery survey in May–July 2025. Institutional data were deliberately collected after the student survey in order to map the structures in place. We have revised the Methods section to clarify this distinction. The temporal offset between data sources was already acknowledged as a limitation.

4- please explain about the validity and reliability of all tools used for data collection.

Thank you for this helpful suggestion. We have expanded the Methods section to clarify the development, validity, and reliability of each data collection instrument.
The student questionnaire was developed iteratively from existing instruments (Schoenefeld et al.) and piloted; it underwent pilot testing. Although the questionnaire has not undergone formal psychometric validation, its development was informed by previously published instruments and pilot testing supported its content validity.
For the deanery questionnaire we describe the item development and content basis. For the website analysis we report inter-rater procedures: extraction by a trained researcher with independent cross-validation of a subset and consensus resolution of discrepancies. The website analysis search strategy is also described in detail in supplement 2.

5- “For analyses at the faculty level, only faculties with at least 50 completed student survey responses were included.” Why?

Thank you for pointing this out. The threshold of 50 completed questionnaires was chosen to ensure that faculty-level estimates were based on a sufficient number of respondents to provide reasonably stable descriptive estimates and to minimize the influence of random variation resulting from very small sample sizes. We have clarified this rationale in the Methods section. Faculty-level estimates from very small local samples are unstable and dominated by sampling noise. The ≥50-response threshold reduces the influence of small-cell variability and yields more reliable institution-level estimates, while we acknowledge it excludes smaller faculties.

6- how about the ethical considerations?

Ethical considerations are reported in the Ethics Statement: “The study protocol was reviewed by the Ethics Committee of the Medical Faculty of Julius-Maximilians-Universität Würzburg. The committee determined that the study did not require formal ethics approval because no identifying personal data were collected and participation was voluntary and anonymous (waiver issued on 11 December 2023; reference: 20231204 01). All data were collected in accordance with applicable data protection regulations and in coordination with

the institutional data protection officers. Student participation was voluntary and required informed consent to the privacy policy. The faculty questionnaire collected institutional information only.”

<u>Results</u>
1- in table one some percentages are more than 100%!

Thank you — this needed clarification. The sub-items under “dedicated counselling services” (university / medical faculty / university hospital) and under several other rows are multiple-response items: a single faculty may offer a service at more than one organisational level, so column sums exceed 100%.

2- what does it mean by “Evaluation” in table 1.

“Evaluation” referred to whether the faculty had ever formally evaluated the effectiveness of its counselling service. We have relabelled this row “Formal evaluation of the counselling service ever conducted” to remove ambiguity.

3- “personal experiences of sexual harassment” how were assessed?

Thank you for this comment. We have clarified the assessment procedure in the Methods section. Before answering the relevant items, participants were presented with a standardized definition of sexual harassment based on the German General Equal Treatment Act (Allgemeines Gleichbehandlungsgesetz, AGG), illustrated with concrete examples. Participants were then asked to report whether they had personally experienced sexual harassment during their medical studies and completed follow-up questions on the type, location, perpetrators, frequency, reporting behaviour, consequences, and use of support services using multiple-choice and Likert-type items.

Reviewer 2

Prof. Angela CARTA, University of Verona

Comments to the Author:
The manuscript addresses the important issue of sexual harassment, focusing on medical students. The authors have adopted a mixed-methods approach, conducting a national survey to identify the most significant barriers and provide guidance for future prevention strategies.
There are no particular concerns regarding the article's content or scientific validity, but it would be best to consider certain suggestions before publication.

We thank the reviewer for the positive assessment and the constructive suggestions, all of which we have addressed as detailed below.

On page 3, lines 47–57, it would be advisable to add a concluding sentence on future research.

As suggested, we added a concluding sentence on future research.

On page 5, lines 6–15: To give the reader an idea of the known extent of the phenomenon, it might be useful to add data on prevalence.

Thank you for this comment. We have added national and international data on the extent to more clearly frame the research gap.

Lines 35–40: The aim of the study is clearer in the abstract than here. It would be preferable to rephrase and explain the aim more clearly, bearing in mind that the description of the methodology referred to is, in any case, set out in the methods section. In these lines, the primary objectives and, where applicable, the secondary objectives of your study should be stated more clearly.

Thank you for pointing this out. The aim has been rephrased, and the primary and secondary outcomes are now stated explicitly

Lines 52–54: It would be advisable to include a brief explanation of the 'iterative process' used to develop the questionnaire, together with references to the theoretical sources that guided its development, where applicable.

Thank you for this comment. We have expanded the Methods section to clarify what is meant by the iterative development process.

On page 6, line 31: the reference cited in the text as a source of information on the questionnaire should be revised. Currently, the manuscript cannot be considered a 'previous manuscript', as it is a manuscript under review that has not yet been accepted for publication. It is therefore advisable to provide a brief general description of the questionnaire's main dimensions to facilitate a better understanding of the methodology adopted.

Thank you for this helpful suggestion. This issue has now been resolved because the previous manuscript describing the development, content, and methodology of the student questionnaire has since been published. We have replaced all references to the manuscript as being "under review" or a "previous manuscript" with the full citation (Förstel M, Vogt M, Drossard S. Sexual harassment at German medical schools – a national cross-sectional study. BMC Medical Education. 2026;26:558). In addition, we have expanded the Methods section by including a concise description of the questionnaire's development and its main thematic domains, allowing readers to understand the instrument without having to consult the previous publication.

On page 8, lines 29–34: What is the response rate among medical students? Although 5,691 students constitute a large population, it is important to assess how representative this sample is. Response rates are often low in online surveys on this topic; therefore, it is important to discuss the representativeness and implications of these data.

Thank you for this important comment. Because the survey was distributed through multiple open channels, including the German Medical Students' Association (bvmd), deans' offices, student councils, and social media, it was not possible to determine the number of students who received the survey invitation. Consequently, a denominator-based response rate could not be calculated reliably, and we now state this explicitly in the Methods section. To address the reviewer's concern regarding representativeness, we have also expanded the Strengths and Limitations section. There, we discuss the potential for selection bias inherent to voluntary online surveys, as well as the nationwide recruitment across 44 medical faculties and the large sample size, which support the breadth of the findings while acknowledging that the results may not be fully representative of all German medical students.

On page 9, lines 15–19: a brief explanation (either here or in the 'Methods' section) is required of the categorisation of students into 'non-medical background', 'students in clinical stages' and 'pre-clinical students', and the result should be briefly discussed in the 'Discussion' section.

Thank you for this suggestion. We have clarified these categorizations in the Methods section. Stage of study was operationalised according to the German medical curriculum (pre-clinical: semesters 1–4, prior to the First State Examination; clinical: semester 5 onwards). Prior non-medical vocational experience was defined as the completion of a vocational training programme or university degree outside medicine before entering medical school. In addition, the Discussion already includes an interpretation of these findings, suggesting that the higher reporting odds among students with prior non-medical vocational experience and those in the clinical stage may reflect greater age, prior professional socialisation, and greater familiarity with formal workplace procedures.

On page 12, lines 23–26, a brief comment should be added regarding the data's representativeness. The response rate reported by the medical schools is high and should be considered a strength; on the other hand, as noted above, the response rate among medical students warrants brief consideration in light of comparisons with the literature.

We have revised the Strengths and Limitations section to address the representativeness of both datasets more explicitly. We now highlight the high response rate of the deanery survey (81.0%; 34 of 42 faculties) as a strength that supports the robustness of the institution-level findings. In contrast, because the student survey was distributed through multiple open recruitment channels, a denominator-based response rate could not be calculated. We explicitly acknowledge this as a common limitation of voluntary online surveys on sensitive topics and discuss the potential for self-selection bias, while also noting the large nationwide sample from 44 medical faculties. The survey included responses from 5,681 students, corresponding to approximately 5–6% of all medical students in Germany. We have included this information in the Strengths and Limitations section while emphasizing that this proportion should not be interpreted as a response rate because the number of students who received the survey invitation is unknown.
