# Supplementary material for: Sexual harassment at medical schools: barriers to reporting and strategies for improvement - a nationwide, cross-sectional, multi-method study from Germany
Source: BMJ Open. 2026 Aug 21;16(8):e114374. doi: 10.1136/bmjopen-2025-114374 (PMC13504847; doi:10.1136/bmjopen-2025-114374)
Supplement: Supplementary file 4 [file bmjopen-16-8-s003.docx]

# **Supplement 3: Website Search Strategy and Analysis**

## Search Strategy

### Institutions searched

We compiled a list of all state-recognized German medical faculties using the Medical Faculty Association of Germany (MFT) directory. To ensure completeness, an additional Google search using the terms "Medizinstudium" and "medizinische Fakultät" was performed, identifying a total of 46 German institutions offering undergraduate medical education (including public and private/partner institutions).

The list was subsequently cross-checked against the participating institutions in the nationwide student survey. As the documentary analysis was designed to complement the student survey, only institutions represented in the survey were included in the website analysis, resulting in a final sample of 44 medical faculties.

### Search Strategy

For each institution, the search began on the official medical faculty website. If no relevant information was identified, the search was extended to the affiliated university hospital website, followed by the parent university website. Additional institutional webpages (e.g. equality offices, ombudspersons or counselling services) were examined only when linked from these primary websites or when necessary to locate relevant information. Public and private institutions were searched using the same procedure.

The search included both webpages and downloadable documents hosted on institutional websites, irrespective of document format. Eligible sources included, but were not limited to:

webpages describing institutional policies or support services; codes of conduct; policies and guidelines; university regulations; reporting procedures; handbooks; downloadable PDF documents; and contact pages for relevant institutional offices. No restrictions were applied with regard to document type. If multiple versions of the same document were available, only the most recent version was included.

Only publicly accessible institutional information was included in the search. Login-protected resources (e.g. intranet pages or learning management systems) were not accessible and were therefore not considered.

### Search Workflow

Each institution was searched using a standardized, predefined workflow. The search was conducted in the following sequence:

1. Review of the official medical faculty website.
2. Manual navigation from the homepage (maximum of ten clicks).
3. Use of the website's internal search function.
4. Review of relevant webpages and downloadable documents identified through the search.
5. If no relevant information was identified, repetition of steps 2–4 on the affiliated university hospital website.
6. If still unsuccessful, repetition of steps 2–4 on the parent university website.
7. Review of linked institutional webpages (e.g. equality offices, ombudspersons, counselling services or legal affairs) when referenced from the primary websites or when required to locate relevant information.

Manual navigation was limited to a maximum of ten clicks from the homepage. This limit was defined a priori because information requiring more extensive navigation was considered unlikely to be readily accessible to students or staff, who would instead be expected to use the website's search function.

The internal search function of each website was used with the following predefined search terms, entered individually:

- *Sexuelle Belästigung*
- *Diskriminierung*
- *Meldestelle*
- *Beratungsstelle*
- *Verhaltenskodex*
- *Code of Conduct*
- *Gleichstellung*
- *Beschwerde*

The selected search terms were chosen to reflect the terminology most commonly used by German universities for reporting structures, equality services and institutional policies related to sexual harassment.

Search terms were applied sequentially. If relevant information was identified, the corresponding webpages and associated documents were reviewed for eligibility and data extraction. If no relevant information was found, the next predefined search term was applied.

### Eligibility criteria

Documents were considered eligible if they

1. explicitly addressed sexual harassment or gender-based harassment/violence, or
2. described institutional reporting procedures or support services applicable to such incidents.

No restrictions were applied regarding document format. Eligible sources included webpages, downloadable PDF documents, policies, guidelines, regulations, codes of conduct, reporting procedures, handbooks and contact pages. When multiple versions of the same document were available, only the most recent version was included.

## Data Extraxtion and Analysis

### Data extraction

Relevant documents were reviewed using a standardized extraction form developed a priori. For each institution, we extracted information on the availability of dedicated counselling and reporting services for medical students, the existence of institutional codes of conduct or policies addressing sexual harassment, the availability of psychosocial and legal counselling, reporting pathways, and the description of complaint procedures, including information on complaint processing and investigation timelines. Variables used for the comparative analyses reported in the manuscript included the accessibility of reporting options, visibility of information on institutional websites, availability of dedicated reporting services, reporting channels, and institutional support mechanisms.

### Data Analysis

The accessibility of reporting services and the visibility of relevant information on institutional websites were assessed using two predefined ordinal rating scales (Tables 1 and 2).

Table 1: Accessibility Rating

| 0 | No dedicated service found |
| --- | --- |
| 1 | Dedicated service available, contact via email/telephone only |
| 2 | Dedicated service available, also reachable via chat/messenger, including anonymous options |
| 3 | Dedicated online platform, allowing anonymous reporting |

Table 2: Visibility Rating

| 0 | No information available |
| --- | --- |
| 1 | Information available, only accessible within embedded documents (e.g. PDF-files, brochures, statutes, etc.) |
| 2 | Information accessible via menu or search function |
| 3 | Information prominently displayed, e.g., on the starting page |

We further evaluated the available reporting pathways, the scope of institutional support services, and the information provided on complaint handling procedures. Specifically, we assessed whether documents described the reporting process, the investigation procedure, and expected timelines for processing complaints.

The content of institutional codes of conduct and sexual harassment policies was analysed using a coding framework adapted from the criteria for effective anti-harassment policies proposed by Blumell et al. [19] (Table 3). Each criterion was coded as present or absent.

Table 3: Criteria of effective anti-harassment policies as proposed by Blumell et al.

**Effective anti-harassment policies**

- clearly define what constitutes sexual harassment
- define both categories of sexual harassment and provide detailed descriptions of specific behaviours that constitute sexual harassment
- identify the wide range of potential harassers, acknowledging power imbalances, and the possibility of harassment from external parties such as patients
- stress “zero tolerance” for sexual harassment
- use gender-neutral language
- stipulate that complainants will not face retaliation

### Data collection and Quality Assurance

Data collection was conducted by one trained researcher using the standardized search and extraction protocol. To ensure consistency and accuracy, a second researcher independently reviewed a random sample of 10 medical faculties using the same protocol. In addition, all institutions for which no relevant information or documents were identified during the initial search underwent a complete independent secondary review. Likewise, all cases in which the primary reviewer was uncertain about the eligibility of a document or the classification of extracted information were independently assessed by the second reviewer. Any discrepancies between reviewers were discussed within the research team and resolved by consensus.
